# A qualitative study exploring stakeholder perspectives on the use of biological samples for future unspecified research in Malawi

**DOI:** 10.1186/s12910-020-00503-4

**Published:** 2020-07-20

**Authors:** Limbanazo Matandika, Ruby Tionenji Ngóngóla, Khama Mita, Lucinda Manda-Taylor, Kate Gooding, Daniel Mwale, Francis Masiye, Joseph Mfutso-Bengo

**Affiliations:** 1 Centre for Bioethics in Eastern and Southern Africa, College of Medicine, University of Malawi, Private Bag 360, Chichiri, Blantyre 3, Malawi; 2 College of Medicine Research Ethics Committee, University of Malawi, Blantyre, Malawi; 3 Oxford Policy Management, Oxford, UK; 4 John Hopkins- One Community Project, Blantyre, Malawi; 5 The Centre for Medical Ethics and Law (Department of Medicine), Stellenbosch University, Tygerberg Campus, Cape Town, South Africa; 6 Directorate of Postgraduate Studies, Research and Outreach, Malawi University of Science and Technology, P.O Box 5196, Limbe, Malawi

**Keywords:** Biobanking, Informed consent, Future use, biological samples and trust

## Abstract

**Background:**

There is growing interest in the collection, storage and reuse of biological samples for future research. Storage and future use of biological samples raise ethical concerns and questions about approaches that safeguard the interests of participants. The situation is further complicated in Africa where there is a general lack of governing ethical frameworks that could guide the research community on appropriate approaches for sample storage and use. Furthermore, there is limited empirical data to guide development of such frameworks. A qualitative study to address this gap was conducted with key stakeholders in Malawi to understand their experiences and perspectives regarding storage and usage of samples for future research.

**Methods:**

This study conducted 13 in-depth interviews with ethics committee members, regulators and researchers, and five focus group discussions with community representatives and clinical trial participants in Malawi. Interviews and focus group discussions were audio-recorded, transcribed verbatim, and thematically analysed.

**Results:**

On the current regulatory guidelines that governs the collection, storage and reuse of samples in Malawi, participants highlighted their different understanding of it, with some indicating that it prohibited the reuse and sharing of samples, while others believed it permitted.

Views on the informed consent model used in Malawi, some stakeholders expressed that the current model limited options for sample contributors regarding future use. Researchers supported storing samples for future use in order to maximize their value and reduce research costs. However, they expressed concern over the exportation of samples highlighting that it could lead to misuse and would not support the development of research capacity within Malawi. They recommended use of broad consent or tiered consent and establishment of biobanks to address these concerns.

**Conclusions:**

Study findings highlighted the need for a review of the current regulatory guideline and the development of infrastructure to support the use of stored biological samples for future use among the research community in Malawi. At the moment, there are ethical and practical concerns arising from the collection, storage and secondary use of biological samples make it hard to reconcile scientific progress and the protection of participants.

## Background

Human biological samples, such as tissues, organs, blood, cells and DNA, have been critical for global advances in biomedical research and population health [1]. For this reason, it has become an acceptable practice in the scientific community that samples and data should be made available for researchers [2, 3].

Samples and data collected in low middle income countries (LMIC) are sometimes exported to high income countries (HICs) for analysis due to a number of reasons including; lack of resources, lack of technical capacity, and lack of infrastructure like laboratories [4]. Increased development in biotechnology creates demand of reuse of samples and data in LMIC. In the past decade, Malawi has experienced tremendous growth in hosting research and researchers, collaborating with sponsors and funders from high Income countries (HIC). This has inevitably led to the collection and storage of samples for future research with some left-over samples exported to HICs for unspecified future research [3].

Exporting and sharing of samples and data from LMICs brings ethical challenges. For example, there are questions regarding specific mechanisms that are employed to safeguard the interests of research participants such as sample donors, [2] and appropriate models of consent for sample storage and reuse. There are also concerns about how to share research benefits in a way that is fair and acceptable to participants, their communities and funders [2–4].

### Biobanking

Biobanks are repositories for the organized collection of human biological samples, with associated personal health information [5]. The establishment of biobanks in LMICs has received significant support during the past decade as it has been viewed as the best way to build local research capacity and facilitate the rapid delivery of research findings. In Africa, biobanks have been promoted by the Human, Health and Heredity in Africa (H3Africa) group [6], which is a consortium of African scientists funded by the Wellcome Trust and the US National Institutes of Health (NIH) in partnership with the African Society for Human Genetics. H3Africa aims to promote genomic research expertise on the African continent with the aim of using genomic methods to address health inequities in both communicable and non-communicable diseases [4]. As part of its work, the H3Africa consortium has supported development of biorepositories in Nigeria, Uganda and South Africa. Setting up biobanks minimizes exporting samples from LMICs to HICS [7]. Samples can be stored and be used in future research in their countries of origin, thereby tackling the ethical challenges associated with exportation of samples to HICs and sample misuse [7]. Overall, genomics and biobanking have shifted the way research is done since it allows openness, data sharing, collaboration of scientists in LMICs and HICs and reuse of samples for future unspecified research [7].

The drive to establish biobanks in Africa is a positive initiative. However, there remain gaps in research governance, oversight practices and regulatory guidance on consenting procedures for the collection, storage and reuse of samples for future research. For instance, a study done in Kenya in 2005 reported that 25% of protocols reviewed indicated the requirement for sample storage and use, but only half of the studies informed research participants about sample storage and future use during the consenting process [2]. These findings point to the ethical concerns related with biobanking including other relevant concerns such as governance, community engagement, international collaboration and sample sharing [8].

### The role of regulatory authorities and ethics committees

National Research Ethics Committees (NRECs) and National Regulatory Boards (NRBs) are mandated to safeguard the rights and welfare of research participants, by ensuring that ethical challenges that are present in research are mitigated and that researchers scrupulously follow ethical principles and frameworks [9].

In Malawi, Research Ethics Committee (RECs) such as the National Health Sciences Research Ethics Committee (NHSRC), the Malawi University of Science and Technology Research Ethics Committee (MUSTREC), the College of Medicine Research Ethics Committee (COMREC) as well as the National Commission for Science and Technology (NCST), which is the national regulatory board (mandated to promote and regulate the conduct of research in Malawi), have adopted policies, guidelines and ethical principles to ensure that research participants’ interests are safeguarded. However, implementation of these policies remain a challenge [8]. In view of this, in 2012 an NCST statement stopped the storage and future use of biological samples for unspecified research. Therefore, this policy statement motivated this research study that aimed at exploring views of key research stakeholders on the collection, storage and future use of biological samples for unspecified research. This study firstly explored ethical issues surrounding the collection, storage and use of human biological samples from LMICs in future unspecified research. Secondly, the study examined views on informed consent models for collection and reuse. Finally, views and perspectives of different stakeholders on biobanking as well its benefits and challenges were documented.

### Aims and methods

This study aimed to explore experiences, views and practices of critical stakeholders (thus ethics committee members, regulators, researchers, community representatives and research participants) regarding sample collection, storage and reuse, as well as consent models currently in use in Malawi. The study covered a 6-month period from May to October 2018.

We conducted a total of 13 In-Depth Interviews (IDI) with purposively selected key informants identified from institutions that are extensively involved in clinical research that collect, store and use human biological samples. We sampled a diverse group of key informants with a purpose of maximizing diversity of research experience and research themes. We conducted three IDIs each with REC members and national regulatory board personnel, and seven IDIs with researchers from six different research institutions in Lilongwe and Blantyre. A total of five Focus Group Discussions (FGD) were conducted in chichewa; three of them with clinical trials research participants from Chikwawa, Lilongwe and Karonga districts and two with Community Advisory Board (CAB) members in Lilongwe and Karonga respectively. Each FGD had between eight to 10 homogenous participants. The number of participants were limited to 10 to ensure active participation [10], refer to Table 1.

**Table 1:** Characteristics of study participants

Data collection method	Group/Key Informant	Number of IDIs/FGDs	Number of participants
IDI	REC members	3	3
IDI	National regulatory board personnel	2	2
IDI	Researchers	8	8
Total participants in IDIs			13
FGD	Research participants	3	24
FGD	CAB members	2	23
Total participants in FGDs			47
Total number of participants in the whole study			60

For FGDs, we approached people that were enrolled in clinical trials at the time of our research and had donated samples as part of their research procedures. Through research sensitization/education sessions, administrative staff and research nurses from the participating institutions informed clinical trial participants about the study and briefed them about our study objectives. Those who voluntarily agreed to participate in the study were recruited and requested to come on the next day for an FGD. To recruit CAB members, respective research institutions first briefed CAB members about the study during their CAB meetings. Following their willingness to participate in the FGDs, CAB members were called for a discussion on an agreed date.

IDIs were guided by open ended questions based on an interview guide that was revised throughout the study in consensus with the researchers following the inductive approach. Questions were revised on the basis of participants’ experiences and responses that required further assessment. Data collection was stopped when saturation was achieved and no more relevant themes were being identified [10].

All interviews were recorded, translated and transcribed verbatim. The interview transcripts were analysed using Thematic Content Analysis [11]. Data was coded with inductive descriptive codes generated from the interview transcripts. Themes emerging from the data were discussed among the investigators and coding differences were resolved by reaching a consensus [12], refer to Table 2. Data was managed with NVivo 12.0.

**Table 2:** A Summary of seven major themes derived from the interviews

Themes	
Experiences and concerns with the current practice of collecting, storing and using samples in Malawi	
Benefits of reusing samples	
Perception on biobanking	
Ethical Challenges of setting up biobanks in Malawi	
Views on current informed consent models	
Concerns over specific informed consent	
Views on research governance and oversight	

College of Medicine Research and Ethics Committee (CoMREC) reviewed and approved the study. Its ethics reference number is P08/17/1233. Institutional permissions were sought from all participating institutions and their letters of support were submitted to the CoMREC prior to the interviews, written individual consent was obtained from all research participants who participated in either the FGDs or IDIs.

## Results

Each of the theme is discussed below.

### Experiences and concerns with collection, storage and use of biological samples for research

IDIs with researchers and REC members revealed a number of experiences regarding collection, storage and use of biological samples for research in Malawi. Both researchers and REC members acknowledged that the current practice does not permit the collection of biological samples for future research purposes as explained by the participant below:

*"The current policy is to discard the leftover sample. The collection, and the shipment can still be done but under a material transfer agreement. After all that, after a specific period, let’s say the first 5 years, discard, although l personally have problems with that. l would say it was a wrong practice because that was not a regulated policy, it’s simply a practice". (IDI 234, Regulator).*

In addition, the practice of sending samples to institutions outside Malawi for further analysis was a concern. REC members concurred with researchers on concerns about their inability to control what happens to exported samples as expressed below.

"*… since we do not have control for operations happening outside the country but we know that some samples are still being kept coming from Malawi under the pretext of lack of capacity in Malawi, but these are research institutions that have been here for some time, some more than 20 years, 25 years, but still more there is no capacity to conduct some tests. Yes, some are complicated and will call for exportation but some are not complicated and by now Malawi could have had capacity to manage or do tests on those samples". (IDI 221, REC Member).*

It was further revealed that fear that samples may be sold and misused were some of the reasons that triggered the release of the guidelines stipulating Malawi’s stand on prohibiting the use of biological samples for future research.

"… . *people have no idea why they are collecting the specimens and sometimes they can sell those specimens or use to discover something, make money out of it, patent it, and not acknowledge where it come from and ripe all the benefits. (IDI 232, REC Member).*

Another concern was lack of clear guidelines to offer guidance to researchers on how to destroy left over samples that are shipped outside of Malawi.

"… *even the specimens that are collected and sent outside, we fill an MTA (Material Transfer Agreement), investigators fill-in MTAs but there is no indication in the guidelines. Material transfer agreements are not actually covered in the guidelines. They are there, just documents to fill-in. And so the investigator sends specimen outside, they promise to destroy and there is no follow-up to say have those specimens been destroyed. How? We just believe that ok they will be destroyed? The guidelines are silent on that (IDI 232, REC Member).*

### Benefits of re-use of samples

Researchers expressed support for reuse of samples for future unspecified research by specifically indicating that such practice helps to lessen the cost and time burdens on researchers.

*"I tell you, it can be better for master students, everybody wants to collect fresh data, yet it is very expensive. We are poor in Malawi and some other bright people fail to do good studies because they are looking for money to go recruit people to collect samples. If we had a biobank associated with the data for those individuals, I think our master’s students and PhD students would look at what data is there and analyze it or maybe do one additional test and they would have the other results ready. I think it’s important for Malawi. It would actually make our research more efficient and get more people do more research by using available data and samples" (IDI 224, Researcher).*

*"The benefits are that they allow you to use data from a defined clinical trial cohort to better leverage information from that cohort. You wouldn’t be able to ever recreate that same cohort because it is expensive". (IDI 226, Researcher).*

### Perceptions on biobanks

REC members, researchers and research participants were asked about their perceptions on the establishment of a biobank in Malawi. Most of them were concerned about access to samples by other researchers and how feedback to the community would be provided. Additionally, REC members and researchers expressed concerns over the lack of proper consenting guidelines on sample ownership and poor governance frameworks to support the monitoring of researchers who collect and store samples.

"*… we need to have access to the storage facility for us to know how the samples are kept. Furthermore, the results need to be given to the participants. They may collect samples but they have to know that feedback is very important. You need to be aware of any problem/disease that you have been diagnosed with for you to take action". (FGD 441 with Research Participants).*

One REC member’s concern about ownership of collected samples was put like this:

"...*of course the other tricky thing is who owns these specimens if the patient has donated or you have collected those samples? … is it Malawi government, is it the research, it is the research institution, it is still the patient in such a way that you have to come back to the patient to ask for another consent for future research? In the guidelines and in the framework, there has to be an issue to do with ownership." (IDI 221, REC Member).*

The concern expressed above is related to the lack of clear consenting guidelines in Malawi for storage and future use of samples. One researcher echoed this concern stating that there appears to be no ethics governance in place to compel researchers to declare what samples they have kept and what samples have been destroyed.

*"I think in my experience, there has never been a time where someone has come to me to ask for sample that I collected for some study and ask me where the samples are. I think the ethics committee believes that after five years have gone, then we have destroyed the samples. I must say that there is no kind of inspection to see that indeed the samples were destroyed". (IDI 224, Researcher).*

The above concerns point to the ethical concerns related to governance and oversight of stored samples.

### Ethical challenges of setting up biobanks in Malawi

Researchers, REC members and study participants were asked about the ethical challenges of setting up biobank in Malawi. Study participants indicated an interest to know how incidental findings will be communicated back to them and medically acted upon. Research participants echoed that by providing biological samples, researchers are placed with a duty to keep them informed on any issues related to their health that will be identified at a later stage or in future research.

*"In my opinion, they have tested maybe cough, or headache, they should tell me what disease has been found in me. The same when they have tested you for any disease, they should tell you". (FDG 440, Clinical trial participants).*

Study participants questioned why samples could be kept for future research without much details to share with them on how their samples would be used*.*

*"My fear is taking my blood today and use it next year". (FGD 440, clinical trial participants,)*

"*… same with me, the period between blood giving and use is long, so we would want to hear concrete reasons why they are failing to tell us the actual reason". (FGD 440*, *clinical trial participants,).*

*"...but also, we don’t get it when they say they are going to use it after five years, will it be to our knowledge or not?" (FGD 440, clinical trial participants).*

Researchers also highlighted the ethical concern of sample sharing with other researchers. It was reported that there is need for proper guidance on sharing and access to samples.

*… "What it takes to have your samples going to a biobank for example, how long the samples can be kept for and ahhh you know at what point those samples are to be available to other researchers" (IDI 222, Researcher).*

CAB members cautioned researchers on some important considerations that ought to be followed for ethical storage of their samples for research purposes.

*"They should be mindful that the samples belong to the participants in a certain area; as such they have to exercise confidentiality. They do not have to publicize our results". (FGD 441, CAB members).*

### Views on current informed consent models

The study also examined what respondents regarded to be the most appropriate way of obtaining informed consent for secondary collection, storage and reuse of samples for future research purposes.

According to researchers and REC members, specific informed consent is permissible in Malawi. Researchers reported having been specific during informed consent processes by outlining what tests will be done in order to answer the current approved research objectives. However, some researchers reported challenges in obtaining specific informed consent. For instance, in pediatric and autopsy studies obtaining informed consent procedures is very challenging as some guardians consent while distressed. Researchers described informed consent procedures conducted during this time as very critical hence the need to use dynamic consent in order to "*maximize the return on the informed consent by answering as many scientifically relevant questions as possible from a single case" (IDI 223, Researcher).* Samples collected from children and autopsy studies were regarded as precious resources taking into consideration the nature of the consenting process and scarcity of such cases.

"*We always have our informed consent form. Maybe I should take a step back and say that the samples that we often store for future use are from autopsies. … … … somebody’s baby just died and you are asking them to do an autopsy so it’s difficult to get consent, and you know we want to maximize the return on that consent and scientifically we want to get as much as possible out of that one case. … We always try to be specific in our questions. (IDI 223, Researcher).*

### Concerns over specific informed consent: Can’t we just keep the samples?

Most researchers argued that even though Malawi has restrictive policies on sample use for future research, the practice of collecting samples and keeping them with the expectation of using them for other research purposes in future is ideal as such samples are regarded as very precious resources. They noted that samples could be used for both academic and public health reasons in Malawi, hence, keeping for re-use would maximize their utility. However, the current specific informed consent model restricts the use of samples beyond the current study objectives.

"The *thing is, it doesn’t allow you to do that. They will give you consent only for the current approved study. After you are done with that you discard them. You do not keep them but if you haven’t finished, you can store them for only 5 years. If you haven’t finished by that time because you still have some things to do, within the current approved protocol, you can also seek another approva … (IDI 230, Researcher).*

There were views from the REC members that were contrary to those of the researchers on the need to reuse already collected samples. REC members recommended the decision to disregard use of samples for other objectives beyond the current approved study objectives.

"...*it is a matter of safe guarding those specimens isn’t it, so that people don’t use them anyhow (IDI 230, REC member).*

Research participants reported that it was the obligation of researchers to respect their autonomy as arrangements for sample sharing and re-use are agreed upon during the informed consent process. However, study participants felt the issues of confidentiality are very central and place the responsibility to maintain privacy and confidentiality on the researchers who collect samples. The study findings reveal that while study participants may understand the reuse of storage of samples, confidentiality was a top priority to them.

*"As per communication, they said that when samples are donated, they get them to the laboratory for testing. And they exercise extreme confidentiality. (FGD 442, Clinical Trial Research Participant).*

This finding supports REC member’s concern that broad consent undermines participant autonomy.

*"..I have to consent to something that l fully understand and l am aware in terms of what it is and its usage. And you are asking me to give you my consent, my agreement if l am to use that kind of term, to something l don’t know just for future, I don’t know what you are going to do, to what l am giving out to you. And you are saying you are obtaining consent. That is not true, that is cheating, and it’s a lie (IDI 234, Regulator,).*

Some Researchers regarded the current consenting model as inappropriate as it restricts study participants’ rights to consent for future use of their biological samples. REC members expressed concerns about destroying biological samples after single use that were contrary to current stipulated regulations. REC members considered a few scenarios that may require reuse, and stipulated that the prohibition might hinder the promotion of science and that it fails to recognize the harm done to research participants who donated the samples.

"*I mean for the time being the problem is; let’s say you have analyzed your data, you have done your research and you have disposed your specimens, but your findings are suggesting that you could have looked at this and that but you have gotten rid of the specimens. This time you need to write another protocol and collect new specimens and that could be a problem. (IDI 230, Researcher).*

### Views on research governance and oversight

*"..Build local capacity and enhance oversight: Science is evolving, therefore let us value the human samples" (IDI232, Researcher).*

The study also sought stakeholders’ views on governance and oversight of samples that have been collected. Researchers and REC members expressed concerns on some of the challenges and fears faced by both local researchers and research oversight members that triggered the prevention of collecting and reusing of samples for future research. Potential concerns included fear of exploitation of research samples when they have been sent to other collaborators or researchers in other settings away from the primary collecting points, lack of equipment or supplies to conduct robust and comprehensive tests locally that necessitates exportation of samples. Both researchers and REC members expressed perceived feelings of mistrust towards researchers and collaborators.

*"… and I think that’s even true for researchers. I can not 100% guarantee that the samples I am sending, if I am not there they will be used for this particular thing. I would rely on the trust of my colleagues. Ethically you are always worried that if my colleague or if my collaborator is not honest enough they may want to use samples for something else that I do not know. (IDI 222, Researcher).*

Mistrust was reported in three categories; i) between researchers obtaining consent and researchers in custody of the samples after exportation, ii) between researchers obtaining consent and institutions in custody of the left over samples and iii) between study participants and research institutions in custody of the samples.

*"Because I really do push for all the samples to be analyzed here and I can name you researchers here well am not going to but I could who I feel are almost like pharmacists, which is bad. I mean they collect samples here and somebody orders from the US and they say, I have got that I will send it to you. That is completely inappropriate, the researchers here are just like middle men and they are doing nothing for infrastructure, they are doing nothing to train local Malawians, they are not bringing in the machines* etc. *they are just collecting samples and sending out. … …*. *There is really very few assays that I fell we can’t do here at []., I mean I can count on one hand the number of assays that I feel need to be done outside, (IDI 223, Researcher).*

In responding to a question on how issues of mistrust could be addressed, almost all interviewees proposed building local human and infrastructure capacity to conduct tests locally, promotion of good research practices by research institutions, establishing legal and ethical frameworks to provide guidance on sample handling, participatory engagement in the review and revision of ethical guidelines, promotion of effective research governance and introduction of a legal stance on research misconduct.

*"I must admit I find it quite difficult, but I haven’t looked at the more comprehensive guidelines from the NCST. It’s not easy to access them. I think the statement about duration of storage and what to do with the samples after that is too prescriptive and really does not appreciate the value of these human samples in the context of moving science forward so my view would be that if we are going to draft any future guidelines we should look at the trend at which science is going and we should value the human samples that we collect". (IDI 222 Researcher, Southern Region).*

Nevertheless, researchers cautioned that while it is their responsibility to safeguard the quality of biological samples and ensure informed consent, research oversight bodies have a core responsibility to serve as custodians of biological samples collected from research. It was widely recommended that research oversight bodies must engage with relevant research stakeholders during policies and guideline formulation.

*"Consultation processes can be done, maybe coming up with a committee which could look at this. a committee can help because they are going to be looking at the internal capacity of Malawi as well as external challenges or balancing the two between our capacity if we do not have that capacity what can we do, how can we handle samples (IDI 221, REC Member, Central Region).*

These responses reflect the ethical and practical issues that are inter-related to all research stakeholders in Malawi. The solutions and concerns further highlight the absence of the social relationship between REC members and researchers in working towards the common goal of advancing science whilst at the same time achieving high ethical standards. Lack of comprehensive and robust strategies to offset various ethical concerns remain unaddressed.

## Discussion

This study revealed a wide range of ethical and practical issues that emerge from the current practice of collecting, storing and reusing biological samples for additional future research purposes in Malawi. Challenges and opportunities of the current informed consent models and current guidelines were also discussed. The study revealed respondents’ important divergent views based on their roles and obligations and their years of experience in the research community (for example, a REC member, a regulator or researcher). It was also further noted that study participants had differences in opinion based on their experiences and relationship with research institutions.

### "… They are precious resources … so you want to maximize their consent …"

In our analysis, almost all stakeholders widely supported the practice of collecting, storing and reusing samples for future research purposes. This is consistent with existing literature [1, 3, 13, 14]. As mentioned earlier, there is increase in biomedical research activities in Malawi. However, this increase has been accompanied by a large volume of biological samples and data being exported to developed countries for analysis and future use [15] instead of having such biological samples and data stored in Malawi for future research.

In this study, the benefits of reusing samples were categorized into two: 1) reuse of samples offsets costs of conducting primary research. Researchers widely supported this idea as they felt that it is very cost-effective in a setting with limited human and financial resources and 2) remnant samples were regarded as great resources, useful during public health emergency research. This category was widely supported by researchers and REC members.

### Exploitation and research participants protection

Concerns about the restrictive regulations and guidelines that do not permit the reuse of samples for future research has resulted in storage of samples by various institutions with an expectation that guidelines maybe revised and samples could be re-used. This is reportedly a practice widely exercised by research institutions in Africa [7] in spite of the samples not being reused in future research. This is against the principle of maximization of benefits and values for biological samples and data collected from research participants, defeating the principle of cost-effectiveness.

This study suggests that the regulation to prevent re-use and collection of samples for future research is linked to the overall fear of exploitation, lack of trust and transparency and engagement within the research community, coupled with the uncertainty about what the regulation requires in Malawi. Evidently, it is worth noting that these problems are largely systemic and broadly cultural and historical to some extent due to the export of samples. To address these concerns, many stakeholders proposed the need to establish a national biobank and an ethically robust governance framework for biobanking. With more sensitization and training about the scientific as well as ethical merits of longitudinal storage of biological samples, and preserving such collections within the country at institutional or national level, there is chance that these precious resources of donated biological samples will be maintained and used to promote the health and welfare of local residents.

The ethical and practical concerns expressed in this study are in agreement with literature on various perceptions of sample collection, storage and reuse for future research in Africa [1, 3, 13, 16]. As stipulated by various authors, these concerns reveal how low and middle income settings such as Malawi are struggling with development of guidance documents that respond to ethical issues emerging from advances in science and technology, and also development of fair and effective collaborations.

The findings of the study highlight the dilemma among stakeholders in Malawi in balancing the protection of study participants and the need to advance science. It is clearly evident that most stakeholders recognize the importance of reusing samples but the historical and emerging ethical issues are a stumbling block. The responses clearly indicate that there are currently no approaches to safeguard the welfare of research subjects apart from the informed consent models and research oversight responsibilities. This raises issues of respect for autonomy and protection of study participants as ethical issues that require serious consideration in determining roles and responsibilities and defining obligations that come with every role in protecting study participants.

### Informed consent models for future use of samples

The findings of the study highlight key challenges to safeguarding the rights and welfare of research participants regarding the appropriate informed consent models for the collection, storage and reuse of samples for future research purposes. For example, some researchers strongly expressed concern on the use of broad consent while on the other hand REC members strongly refuted the use of broad consent but rather opted for a more generic specific informed consent model that gives study participants capacity to consent for specific future use or to opt out. This finding is in agreement with research conducted by Jantina et al. [7] who stipulated that broad consent guidance documents in Malawi are non-permissible. In view of this lack of permission for broad consent in the regulations of Malawi, we recommend a revision of the current guidelines and regulations to allow broad consent in its current research practice and to be consistent with current research practice worldwide. This recommendation is highlighted in the recommendation section of this paper.

Most study participants were supportive of specific consent while some indicated the need to be re-contacted for future use of their biological samples and data. Most study participants also recommended that they should receive feedback on any incidental findings pertaining to their wellbeing that are medically actionable in Malawi. This contrasts with a study conducted by Wendler et al. in Uganda who reported that most participants supported that they should not be re-contacted for future use of their biological samples and data thereby supporting broad consent for collection of biological samples and data [17]. This study did reveal that study participants were supportive of broad consent on condition that confidentiality of their results will be assured and they gave this responsibility to the researcher.

Our study highlights an important policy requirement for researchers to provide feedback of research findings to research participants. The community engagement guidelines in Malawi require researchers to disseminate research findings to research participants, their communities and policy makers. Study participants hinted return of research results is an important benchmark for engaging research participants, for promoting trust and transparency and for demonstrating respect for persons among prospective sample donors. Promising to return research results as a part of consent process provides “return of value” to sample donors and it is an essential aspect of a robust governance framework.

The use of the current informed consent model that protects study participants from future unknown harm was seen as both unethical and practically problematic for settings with limited resources such as Malawi. Lack of guidelines and reuse restrictions were similarly seen as unethical and related to prohibiting an exercise to personal autonomy as previously reported in Malawi [3]. However, in this study, study participants do trust researchers to abide by various good research practices outlined in informed consent procedures, for example, issues of confidentiality and sample use.

The findings of this study reveal concerns among REC members and local researchers on the fate of samples collected and shipped outside the country since both REC members and local researchers do not have control over samples that have crossed the borders. The concerns allude to the fact that bad research practices cannot disappear in cross-border collaborations especially where local RECs and researchers lack capacity to oversee samples that are exported in collaborative research. It was reported that bad research practices could be dealt with by participating institutions establishing good research practices and policies that promote the welfare and rights of research participants and giving ownership rights to local researchers and local RECs where biological samples are collected.

## Conclusion and recommendations

In conclusion, this study has highlighted various ethical and practical concerns arising from the practice of collection, storage and secondary use of biological samples in future research. However, there are ethical and practical issues that need to be underscored between researchers and research oversight bodies in Malawi. Broadly speaking, most stakeholders supported storage and future use of biological samples. This was on the assumption that current regulations on informed consent models and future use of biological samples would be revised holistically to take into consideration opinions of all key stakeholders, including community members and current research oversight systems which have been developed to cope with the roles and responsibilities of protecting the welfare of research participants that contribute biological samples. Enhancement of research governance, building of social relationships and participatory engagement approaches to developing guidance documents are significant in helping the research community understand values and roles each research stakeholder upholds in order to ensure protection of study participants and advancement of science. It is also imperative that research institutions value good research practices and account for high ethical standards between researchers and research oversight bodies in Malawi. Furthermore, the results indicate that strategy that have been widely known as fundamental to human research protection such as specific informed consent require supplementary strategies to be developed.

It is high time that current regulations on future use of biological samples and consent models in Malawi were revised to maximize the cost-effectiveness and benefits of biological samples collected from Malawian research participants, and to be consistent with current accepted research practices worldwide. Therefore, we recommend the revision of the current regulations in Malawi to allow use of a broad consent in research practice. The permission will call for the education of the general public about the value and importance of broad consent which will eventually lead to the establishment of the first national biobank in Malawi where researchers can deposit remnant tissue samples after first use. The establishment of such a biobank will require development of governance structures similar to those developed by the H3Africa. A potential source of such funding is the H3Africa Consortium which support establishment of biobanks in Africa through the Wellcome Trust and the National Institutes of Health.

## Data Availability

Permission to share transcripts and all data generated during this study was not sort from study participants.

## Abbreviations

CoMREC: College of Medicine Research Ethics Committee
FGD: Focus group discussion
IDI: In depth interview
KI: Key informant
REC: Research Ethics Committee
NHSRC: National Health Sciences Research Ethics Committee
NCST: National Commission for Science and Technology
LMIC: Low Middle Income Countries
HIC: High Income Countries
NREC: National Research Ethics Committee
NRC: National Research Council
MUSTREC: Malawi University of Science and Technology Research Ethics Committee
